# O.2.3-3 Are we working (too) comfortably?: developing and testing an evidence-informed toolkit to reduce sedentary behaviour when working at home

**DOI:** 10.1093/eurpub/ckad133.129

**Published:** 2023-09-11

**Authors:** Sarah Morton, Claire Fitzsimons, Eva Coral-Almeida, Divya Sivaramakrishnan, Ailsa Niven

**Affiliations:** University of Edinburgh, UK; sarah.morton@ed.ac.uk; University of Edinburgh, UK; sarah.morton@ed.ac.uk; University of Edinburgh, UK; sarah.morton@ed.ac.uk; University of Edinburgh, UK; sarah.morton@ed.ac.uk; University of Edinburgh, UK; sarah.morton@ed.ac.uk

## Abstract

Covid-19 permanently changed how desk-based workers spend their day, with many working at home (W@H) some/all of the working week. W@H is associated with increased levels of sedentary behaviour (SB), when compared to office sitting. There are adverse mental and physical health outcomes associated with prolonged SB. A solution is needed to support those who W@H to move more across the day, reducing the negative effects of SB.

Informed by a rapid review and focus groups with desk-based workers, we developed a ‘Toolkit’ comprising educational information about SB, suggestions for reducing SB, and action planning resources. ‘Toolkit’ components were grouped into themes and delivered over 4-weeks by e-newsletter: 1) Move During Meetings; 2) Active Breaks; 3) Home-to-Home Commute; 4) DIY Tech. Scottish Government employees tested the ‘Toolkit’ and provided feedback to allow us to refine ahead of wider rollout.

Staff reported enjoying using the ‘Toolkit’ and found the weekly e-newsletters were a useful reminder to move more when W@H. Suggestions were considered practical, low-cost, and didn’t require additional resource (e.g. buying equipment) to engage with. In terms of refinements, staff felt it would be useful to have organisational support for permission to take breaks when W@H and that there were occasions when it could be difficult to implement suggestions, e.g. during meetings, when busy, and limitations of job requirements. Each participant had their own favourite strategy. The ‘Toolkit’ could be further enhanced through options to personalise. The Active Break week was the favourite theme with 39% of final evaluation respondents indicating preference for this. DIY Tech week was the least favourite (11%).

W@H employees are aware of the risks of prolonged SB, and want to reduce this behaviour without impacting on job role. Validation from employers will nurture a culture of permission to move, however resources providing employees with individualised suggestions of when to move and what to do will be imperative to encourage long-term behaviour change. Through our research, we have developed and tested an intervention ‘Toolkit’ for supporting those W@H to reduce SB. The next stage is to explore AI-based opportunities for personalisation and rolling out more widely.

